# The potential immunological mechanisms of sepsis

**DOI:** 10.3389/fimmu.2024.1434688

**Published:** 2024-07-08

**Authors:** Xinyu Zhang, Yujing Zhang, Shiying Yuan, Jiancheng Zhang

**Affiliations:** ^1^ Department of Critical Care Medicine, Union Hospital, Tongji Medical College, Huazhong University of Science and Technology, Wuhan, China; ^2^ Institute of Anesthesia and Critical Care Medicine, Union Hospital, Tongji Medical College, Huazhong University of Science and Technology, Wuhan, China

**Keywords:** sepsis, immune cells, cell death mode, machine learning, biomarkers

## Abstract

Sepsis is described as a life-threatening organ dysfunction and a heterogeneous syndrome that is a leading cause of morbidity and mortality in intensive care settings. Severe sepsis could incite an uncontrollable surge of inflammatory cytokines, and the host immune system's immunosuppression could respond to counter excessive inflammatory responses, characterized by the accumulated anti-inflammatory cytokines, impaired function of immune cells, over-proliferation of myeloid-derived suppressor cells and regulatory T cells, depletion of immune effector cells by different means of death, etc. In this review, we delve into the underlying pathological mechanisms of sepsis, emphasizing both the hyperinflammatory phase and the associated immunosuppression. We offer an in-depth exploration of the critical mechanisms underlying sepsis, spanning from individual immune cells to a holistic organ perspective, and further down to the epigenetic and metabolic reprogramming. Furthermore, we outline the strengths of artificial intelligence in analyzing extensive datasets pertaining to septic patients, showcasing how classifiers trained on various clinical data sources can identify distinct sepsis phenotypes and thus to guide personalized therapy strategies for the management of sepsis. Additionally, we provide a comprehensive summary of recent, reliable biomarkers for hyperinflammatory and immunosuppressive states, facilitating more precise and expedited diagnosis of sepsis.

## 1 Introduction

Sepsis is a life-threatening organ dysfunction caused by a dysregulated host response to infection (1). Sepsis is a major cause of worldwide morbidity and mortality (2). A national-level observational study assembled epidemiological data on sepsis in mainland China, showing that sepsis affects one-fifth of patients in the intensive care unit (ICU), with a 90-day mortality rate of 35.5% (3).

Due to the heterogeneity of sepsis, the management of septic patients relies on rapid identification and ICU-based life-supporting, including fluid resuscitation, appropriate antibiotics, and emergency resuscitation (4). Following the unsatisfactory clinical results of anti-inflammatory medicines for sepsis, researchers have revisited the investigation strategies for appropriate immune-boosting therapies (5). Several immunomodulatory enhancement drugs have shown benefits in animal experiments and clinical trials, including Thymosin α1, Nivolumab,and so on (6, 7). The continuous immunosuppression of sepsis enhances the longer-term risk of secondary infections and readmission rates (8).

The comprehension of sepsis began from the hyper-inflammation to the realization of the indelible role of immunosuppression, and now the appearance of precise endotypes. In this review, we discuss in detail the role of immune cells, organs, and cell death patterns in the complex organismal homeostasis of sepsis. We also try to explore the future direction of sepsis diagnosis and therapy in conjunction with modern technological developments, i.e., combining with artificial intelligence to excavate multi-omics data.

## 2 Violent restructuring of immune cells

Immunocytes are dramatically altered during the initiation and progression of sepsis. Leukocytes (neutrophils, monocytes, macrophages, natural killer cells, etc.) burst into “cytokine storm” to clear pathogens, while the over-inflammation impairs the stability of the immunological environment (9). The adaptive immune cells, on the other hand, are ineffectively activated and numerically reduced in persistent septic immunosuppression, and suppressive immune cells dominate in survivors. Hence, different immunocytes have central roles in the hyperinflammatory and immunosuppressive phases of sepsis.

### 2.1 Innate immune system

#### 2.1.1 Neutrophils

Neutrophils, the hub of the “cytokine storm”, release high levels of pro-inflammatory cytokines and proteases in the initial phase of sepsis (9). During sepsis, neutrophils originating from bone marrow (BM) experienced an expansion and consisted of immature and delayed-apoptosis neutrophils (10). Apart from inflammation burst in the early stage of sepsis, neutrophils continually present impaired capacity of chemotaxis and antibacterial, reduced oxidative burst, decreased intracellular myeloperoxidase, and excessive neutrophil extracellular traps (NETs) (11). Programmed cell death ligand 1 (PD-L1)-expressed neutrophils in sepsis inhibit T cell activation, induce T cell apoptosis, and stimulate regulatory T cell proliferation by direct connection (11). A whole blood single-cell map (272,993 cells, n = 39 individuals) of sepsis has demonstrated that immunosuppressive neutrophils partly form the functional basis of the extreme response endotype of sepsis (12). This map offers opportunities for personalized medicine.

NETs are a double-edged sword in sepsis, contributing to pathogen clearance while over-released NETs induce endothelial damage and multiple organ dysfunction syndrome (12). NETs were composed of antimicrobial proteins including myeloperoxidase, histones, and a meshwork of chromatin fibers. Histones cause thrombosis (13), while cell-free DNA extruded from NETs can stimulate clot formation and impair fibrinolysis (14). NETosis occurs via suicidal NETosis, vital NETosis, and mitochondrial NETosis (15). Research has revealed that NETosis is associated with other forms of cell death. NETs activated caspase-1-dependent macrophage pyroptosis to enhance inflammatory responses in cecal ligation and puncture (CLP)-induced sepsis in mice (16). Pyroptotic macrophages as well triggered NET formation during both inhaled-lipopolysaccharide (LPS)- and CLP-induced mouse model of sepsis (17). Platelet pyroptosis exacerbates NET formation which releases S100A8/A9 to promote Gasdermin D (GSDMD)-dependent pyroptosis in mice with CLP-induced sepsis (18). GSDMD also participates in NET release and consequent multiple organ damage in the CLP mouse model (19). Autophagy is involved in the formation of NETs during CLP-induced sepsis to protect mice against lethality (20). These referred experiments demonstrate that the detrimental impact of NETs in sepsis extends beyond mere self-compromise, encompassing the demise of other immune cell populations.

#### 2.1.2 Monocytes/macrophages

Monocytes/macrophages play important roles in sepsis-induced endotoxin tolerance (ET) and antigen presentation (21). Long-term endotoxin stimulations (e.g., LPS, bacterial compounds) with monocytes/macrophages impaired their capability to upregulate inflammatory cytokines (e.g., tumor necrosis factor-α (TNF-α), IL-6) when facing secondary attack (21). Additionally, anti-inflammatory cytokine IL-10 upregulated by phagocytes represses the proliferation of T cells as well as fosters the proliferation of immunosuppressive cells as MDSCs (22). Metabolic reprogramming participates in the differentiation of BM-derived immune memory monocytes, resulting in either trained immunity (protective response to secondary infections) or immune tolerance (the maladaptive state characterized by consistent immune paralysis) (23). Immunotolerant monocytes signify heightened vulnerability to secondary infections in septic patients (23). The high expression of NOD-like receptor family CARD domain-containing-3 (NLRC3) is associated with ET, and BM-specific NLRC3 deficiency in mice improves the metabolic reprogramming inducibility of ET in macrophages (24). Long-term ET is dependent on epigenetic and metabolic reprogramming, it is also the key to rescue monocyte/macrophage dysfunction.

Reduced expression of human leukocyte antigen on monocytes is associated with immunoparalysis, a state of immunodeficiency observed in sepsis (25). Also, low monocyte human leukocyte antigen-DR (mHLA-DR) is an acknowledged hallmark of monocyte anergy in sepsis. The decreased mHLA-DR reduces the ability to stimulate lymphocyte proliferation and polarization due to the reduced capability to present antigens to the adaptive immune system (26, 27). Clinically, low mHLA-DR is linked to an increased risk of secondary nosocomial infections and mortality after sepsis (28). Therefore, decreased mHLA-DR levels are highly recommended to assess sepsis immunosuppressive severity (29). In 2020, a single-cell RNA-sequencing decodes the subpopulations of peripheral blood mononuclear cells (PBMCs) from septic patients, identifying a new cluster of monocytes (MS1) (30). MS1 fraction was validated as a classifier for sepsis in 11 datasets, with a summary AUC of 0.9. MS1-B module is related to the anti-inflammatory and subsequently immunosuppressive state. Last year, another unique monocyte subset characterized by HLA-DR^low^S100A^high^ was identified in sepsis, and S100A9^+^ monocytes exhibited immunosuppressive function by impairing the immunity of CD4^+^ T cells (31).

Macrophage polarization is associated with immune homeostasis. The M1 phenotype synthesizes pro-inflammatory cytokines to eliminate pathogens while the M2 macrophage meditates the anti-inflammatory response and tissue repair (32). M2 polarization predominates in late-stage sepsis and increases the proportion of anti-inflammatory cytokines to elicit immunoparalysis (33). Interestingly, macrophages could undergo “re-polarization” due to changes in their microenvironmental stimuli (33). Increasing the proportion of M1 macrophages or decreasing the proportion of M2 macrophages to reach ratio equilibrium has the potential to reverse sepsis-induced immune imbalance (34).

#### 2.1.3 Dendritic cells

The apoptosis of dendritic cells (DCs) is commonly observed in sepsis and their reduction in the secondary lymphoid organs correlates with an increased risk of secondary infection (35). DCs play a crucial role in triggering adaptive immunity. DCs fail to activate T cells is an indispensable component of septic homeostatic imbalance (36). Roquilly et al. demonstrated that DCs turned into immunoparalysis with disabled antigen presentation function and reduced the secretion of IL-12 after the resolution of *Escherichia coli* inhalation-induced primary pneumonia (37). Moreover, the ‘‘paralyzed’’ DCs highly express a transcription factor Blimp, which is linked to tolerogenic functions and outcomes in ICU patients (37). The decreased secretion of IL-12 by DCs contributes to activation incompetence, but rising IL-10 level dampens the differentiation of interferon-γ (IFN-γ)-producing T helper type 1 (Th1) cells and the activation of natural killer (NK) cells in CLP-induced sepsis (38). Conversely, CD8^+^ T cells originating from septic BM elevate the differentiation of DCs with increased production of IL-12 (38). Epigenetically, histone modification enzymes constantly suppress DCs from producing IL-12, persisting for at least 6 weeks after CLP (39), leading to a chronic risk of deadly consequences for the surviving mice. As vital antigen-presenting cells, the population and functional loss of DCs in sepsis compromises the responsiveness of T cells to infection. As reported, Flt3L remarkably improved the number and function of DCs in CLP mice and restored primary CD4^+^ and CD8^+^ T cells (36, 40). Moreover, clinical reports have examined the efficacy of recombinant Flt3L (CDX-301), demonstrating that rescuing DCs could be a promising therapeutic target for sepsis (41).

#### 2.1.4 NK cells

NK cells originate from a similar lineage with innate lymphoid cells but lack the expression of specific T‐cell receptors. This feature benefits rapid, non‐specific immune response for NK cells to intracellular pathogens intrusion (42). In the hyper-inflammatory phase of sepsis, excessive derivation of IFN-γ leads to anomalies in NK cell activation and can initiate cytokine storms through a positive feedback loop, causing severe organ damage (42). One of NK cells’ defects is the main cytotoxic dysfunction, another is the shrinkage of IFN‐γ rendering post-septic patients more susceptible to a second attack (42). Tregs mediate NK cells’ tolerance in sepsis and increased PD-L1 in NK cells contributes to the poor outcome of septic patients (27, 43). The deficiency of NK cells during the immunosuppressive phase of sepsis could be rescued by the adoptive transfer of a homogeneous population or treatment with multiple cytokines such as IL‐2, IL‐12, or IL‐18 (42).

### 2.2 Adaptive immune system

#### 2.2.1 T cells

Lymphopenia is a predictor of both early and late mortality and could serve as a reliable biomarker for sepsis (44). T cells are particularly susceptible to rapid and profound lymphopenia, and their essential role in septic immunosuppression has been firmly established (45). Even if the counts of T cell may recover, the compartment is transformed with significant phenotypic and functional changes (45). The persistent immunosuppressive microenvironment in sepsis causes T-cell exhaustion, as evidenced by defective cytokine production, the loss of memory T cell self-renewal capacity via homeostatic proliferative signals, and the continuous expression of multiple inhibitory receptors (46). Immunosuppressive cytokines, such as IL-10 and transforming growth factor beta (TGF-β), are markedly elevated and induce polarization towards Th2 and Th17 in septic patients with poor outcomes. Persistently increased Th2/Th1 ratio has been demonstrated to correlate with the highest 28-day mortality (47.1%) and incidence of ICU-acquired infections (64.7%) (47). T-lymphocyte dysfunction is implicated in persistent inflammation-immunosuppression and catabolism syndrome (PICS)-related immunosuppression (48).

Immune checkpoint (IC) receptors are specific immune homeostasis membrane molecules expressed on T lymphocytes. In sepsis, ICs induce T cell impotency and exhaustion by providing co-inhibitory signals and attenuating co-stimulatory signals (49). Programmed death-1 (PD-1) is a representative negative regulatory molecular in sepsis, and anti-PD-1 antibody administration has been shown to attenuate T-cell dysfunction and improve survival in CLP-induced sepsis in mice (49). Phase I clinical trials to evaluate the safety and tolerability of PD-L1 inhibitor Nabolutumab and BMS-1 in septic patients have been completed, and further evaluation of PD-1 signal pathway inhibitors is expected (6, 50). In addition to PD-1, other ICs with ligands, including CD28, CTLA-4, OX40, and 4–1BB, have shown great translational potential in animal and preclinical studies (49). Given the transformational value, the intervention of ICs may be effective for the therapeutic exploration of sepsis.

Memory T cells in sepsis survivors exhibit immunoparalysis in reaction to secondary infection and memory T cells’ compartments alter. Jensen et al. identified that memory T cell compartments are biased towards central memory CD8^+^ T cells (T_CM_) in mice with CLP-induced sepsis. The ability of circulatory T_CM_ to address re-infection was impaired relating to the transcriptional landscape and chromatin accessibility change (51). T_CM_ overrepresentation impacts the re-immune ability for secondary striking and interferes with replenishment from effector memory T cells (T_EM_) to the tissue (52).

Unconventional T cells such as gamma-delta (γδ) T cells, mucosal-associated invariant T (MAIT) cells, and natural killer T (NKT) cells are not constrained by antigen recognition of class MHC I and II molecules, they also play fantastic parts in sepsis. The protective functions of γδ T cells in CLP-induced sepsis are shown on bacterial clearance and trigger neutrophil recruitment (53). The reduced adhesion function of γδ T cells in septic patients impairs γδ T cells’ role as professional antigen-presenting cells (54). In clinical, septic patients’ mortality was associated with a significant decrease of γδ T cells (55). With the increasing severity of sepsis, cell death of CD3^+^ CD56^+^ γδ T cells progressively increased, especially in non-survivors (55). MAIT cells exhibited a dramatic decrease in peripheral blood during the early stage of sepsis by streptococcal infections, the MAIT cell compartment may gradually return to normal without re-striking (56). Despite current observation, the mechanism of MAIT cells to protect sepsis remains to be fully articulated. A gene set enrichment analysis has revealed that the early upregulation of IFN-γ following administration of high-dose endotoxemia drives septic immunosuppression (57). This immunosuppression is mediated part by NKT cells as they partially prompt post-septic immunosuppression (57). Several studies highlighted the pathogenic role of invariant natural killer T (iNKT) cells in sepsis and IFN-γ is the central mediator of iNKT cell impairment. In contrast, the protective role of variant natural killer T cells in sepsis is indirectly evidenced by sulfatide, although further contributions remain unclear (56).

#### 2.2.2 B cells

B cells undergo a reduction in septic shock patients. The absolute decrease in immature B cells (IM), naive B cells, and resting memory B cells (RM) is responsible for the increased percentage of the other two subsets, tissue-like memory B cells, and activated memory B cells (58). Lower counts of IM and RM, which respectively act as immediate precursors of mature B cells and immune memory maintainers, indicate a poorer prognosis in sepsis survivors (58). Memory B cell subsets were preferentially depleted among B cells of septic patients (59). In septic conditions, regulatory B (Breg) cells have an increasing circulation number and negatively regulate the innate immune responses mainly by secreting anti-inflammatory cytokines such as IL-10 and promote Treg cell reaction in sepsis (27). CD39^high^ plasmablasts, an expanded B cell subpopulation in CLP-induced sepsis, elevate extracellular adenosine, impair macrophages’ bactericidal activity, and enhance IL-10 production via the A2A adenosine receptor (60). The differentiation of B cells to plasma cells is blocked due to reduced Th1 cells, resulting in decreased immunoglobulin secretion (61). Although not studied as extensively as T cells, excessive apoptosis of memory B cell compartment and amplification of heterogeneous suppressive subpopulations both contribute to sustainable immune disequilibrium in sepsis (59, 60).

### 2.3 Immunosuppressive cells

#### 2.3.1 MDSCs

**Figure 1:**
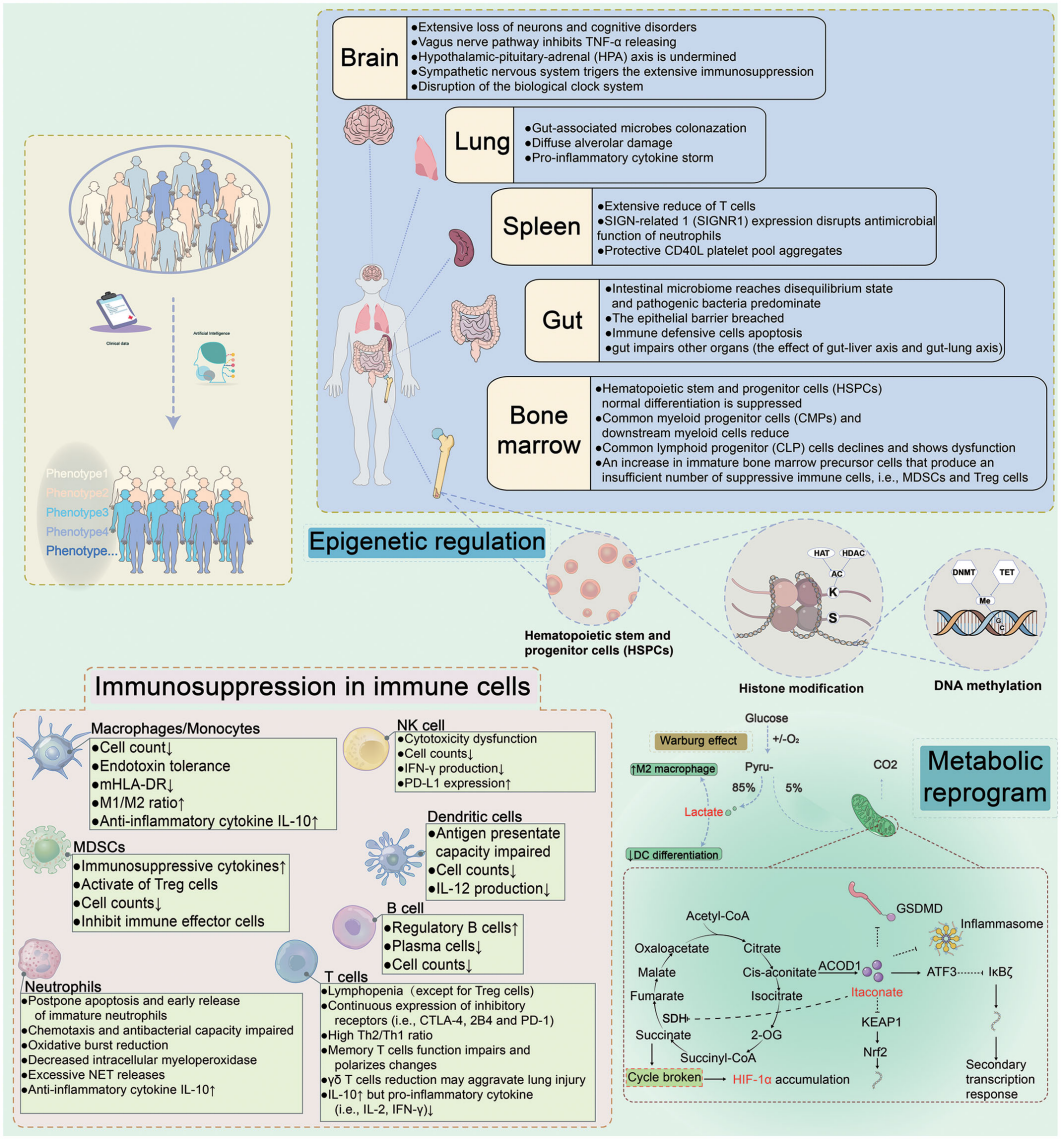
Immune Dysregulation During Sepsis. Sepsis is a sophisticated heterogeneous syndrome. With the development of artificial intelligence, the phenotypes for distinguishing septic patients basing on big data are more accurate. The clinical data of septic patients are gathered and ultimately personalized using artificial intelligence to synthesize the data for precise treatment. Upon prompt identification and classification of septic patients, individualized therapies would be administrated to targeted septic patients. For example, immunostimulatory regimes may be used for immunosuppressive patients, while anti-inflammatory treatments may be effective for septic patients with a hyperinflammatory immunological microenvironment. The underlying mechanisms of sepsis are complex and are interrelated. Different organs (brain, spleen, gut, and bone marrow) contribute to different damage. Metabolic reprogramming and epigenetic regulation in sepsis and immunosuppression present in innate and adaptive immune cells. HAT, histone lysine acetyltransferase; HDAC, histone deacetylase; DNMT, DNA methyltransferase; SDH, succinate dehydrogenase; ACOD1, aconitate decarboxylase 1; ATF3, activating transcription factor 3; HIF-1α, hypoxia-inducible factor 1-α; KEAP1, kelch-like ECH-associated protein 1; Nrf2, nuclear factor erythroid2-related factor 2.

MDSCs are a heterogeneous population that proliferates in sepsis and is comprised of immature neutrophils and monocytes with strong immunosuppressive activity (62). MDSCs can be categorized into granulocytic/polymorphonuclear MDSCs and monocytic MDSCs (M-MDSCs), with the latter subset having more immunosuppressive properties in polymicrobial sepsis (63). The immunosuppression capacity of MDSCs has been demonstrated in several aspects like secretion of immunosuppressive cytokines and activation of Tregs (26). Degradation of L-arginine can damage T cell functions by reducing CD3 zeta-chain expression and the NK cell number during sepsis (62). Although MDSCs are rarely found in healthy individuals, MDSCs are pluripotent when induced by environmental signals, so researchers considered altering the differentiation of MDSCs into mature cells (64). While the early expansion of MDSCs may be beneficial in limiting host inflammation, the prolonged suppression of T cells’ immune responses by MDSCs leads to an unfavorable prognosis (64). Therefore, induction of differentiation maturity or reduced MDSCs from BM in the terminal phase of sepsis is expected to facilitate precision immunotherapy. MDSCs are well-known to induce Tregs amplification, as Lee demonstrated that Tregs could also regulate the immunosuppressive functions and amount of M-MDSCs (65). Crosstalk between these two groups of immunosuppressive cells exists and may lead to immunosuppressive synergy (**Figure 1**).

#### 2.3.2 Tregs

Tregs suppress the proliferation of other effector T cell subsets and divert the phenotypical shift through inhibitory cytokines (27). Tregs also impact innate immunity through the mediation of LPS-stimulated monocytes via the apoptotic Fas/FasL pathway and the direct connection to DCs to downregulate the expression of costimulatory molecules (66). The ratio of Tregs increases in sepsis due to the number of other T cell subsets decreasing. The expression of the anti-apoptotic proteins on Treg makes them more resistant to sepsis-induced apoptosis than other functional T cell subpopulations (66). Th17 and Tregs lie on opposite sides of regulation for the proliferation and polarization of T cell subpopulations, and their ratios serve as reliable biomarkers for predicting sepsis prognosis. The percentage of Th17/Tregs positively correlated with the Sequential Organ Failure Assessment score, as a higher ratio may predict a worse prognosis of sepsis (5). The functions of Tregs vary at different stages of sepsis. The escalation of the Treg cluster may contribute to restoring over-inflammatory eruption during the early stage of sepsis (67). The negative immunomodulatory functions of enhanced Tregs contribute significantly to subsequent sepsis induced-immunosuppression (67).

## 3 Multiple modes of immune cell death in sepsis

**Figure 2:**
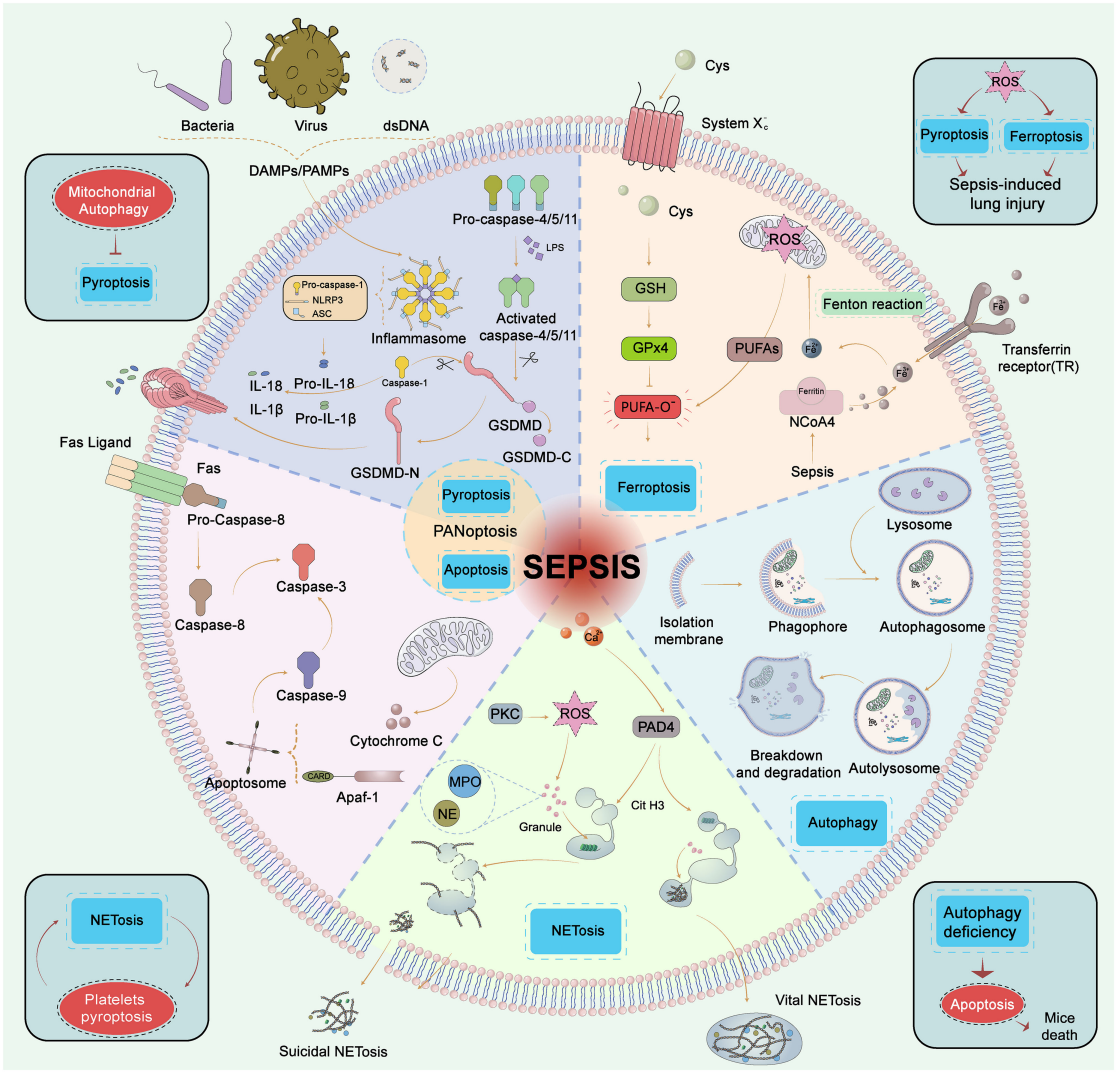
Five types of cell death in sepsis. Pyroptosis, GSDMD is cleaved by caspase-1 (classic pytoptosis pathway) or caspase−4, 5, and 11 (noncanonical pytoptosis pathway) and subsequently integrated into the cell membrane forming a pore. Caspase−1 promotes the release of pro−inflammatory cytokines such as IL−1β and IL−18. Apoptosis, Apoptosis is a programmed process of cell death, which is activated by caspase-8/caspase-3, 9 through extrinsic and intrinsic pathways, respectively. The extrinsic pathway contains the death receptor Fas triggered by the Fas ligand after infection, then Fas activates caspase−8 followed by activation of caspase−3 to trigger the execution pathway of apoptosis. In the intrinsic pathway, cytochrome C is released from mitochondria and then forms an apoptosome with Apaf−1. The apoptosome activates caspase−9 which also activates caspase−3. PANoptosis, PANoptosis is a novel concept in the realm of cell death, that aims to encompass the overlapping and interdependent mechanisms of scorching, apoptotic, and necroptotic apoptosis. Ferroptosis, Ferroptosis is the iron-dependent lipid peroxidation of cell death. The pathways that regulate ferroptosis include both conventional GPX4-dependent and GPX4-independent pathways. GPX4 is reduced in activity and the oxidation of lipids cannot be metabolized by the GPX4-catalyzed glutathione reductase. Subsequent oxidation of lipids by divalent iron ions generates ROS and thus contributes to the onset of ferroptosis. Autophagy, Autophagy is a self-defense mechanism. The autophagosome fuses with the lysosome and further forms a complete autolysosome. Compromised organelles and pathogens are degraded by lysosomal enzymes in autolysosomes and enter the recycling process. NETosis, NETosis is an inflammatory cell death modality of neutrophils and composes of antimicrobial proteins, including MPO, histones, and a network of chromatin fibers. NETosis is activated by alterations in Ca^2+^ and the production of ROS. PAD4 is activated by high Ca^2+^ and drives NETosis via guanylate histones. NETosis was divided into suicidal NETosis and vital NETosis, and suicidal NETosis is accompanied by cell rupture and death, while the vital NETosis is accompanied by cell survival. GSDMD, gasdermin−D; Atg, autophagy−related gene; ROS, reactive oxygen species; PUFA, polyunsaturated fatty acid; GPX4, glutathione peroxidase 4; HMGB1, high mobility group box 1; NE, neutrophil elastase; MPO, myeloperoxidase; PAD4, peptide-arginine deaminase 4; PKC, protein kinase C.

The process of sepsis is intricately allied to the plummet of immune cell count. These cells are eliminated through various modes of cell death such as apoptosis, pyroptosis, autophagy, NETosis, and ferroptosis to eliminate pathogens and clear damaged cells (**Figure 2**) (68). However, the excessive "sacrifice" of immune cells may not necessarily confer a beneficial defensive function for septic patients. Cell death pathways interact with others and prompt the development of sepsis.

### 3.1 Apoptosis

Apoptosis is a programmed process of cell death, activated by caspase-8, 9 through extrinsic and intrinsic pathways to cleave executioner caspase-3, 6, 7 (5). During sepsis, the caspase family was activated significantly, while the expression of Bcl-2 (inhibitor of apoptosis) was suppressed (5). The difference between apoptosis and pyroptosis is that apoptosis would not release inflammatory mediators (69). T cells are over-apoptotic and the immune defensive capability is compromised. Inhibition of lymphocyte apoptosis has been demonstrated to lower sepsis mortality (70). However, delayed neutrophil apoptosis negatively contributed to the severity of sepsis due to the expression of anti-apoptotic factors, and the abnormal long persistence of neutrophils may increase the likelihood of organ damage (71). Altogether, these results indicate that inhibition of apoptosis of mature immune cells may be a strategy for restoring immune function to resist infection.

### 3.2 Pyroptosis

Pyroptosis refers to programmed cell death that is mediated by the gasdermin family, with a prevailing destructive impact on the host during sepsis. GSDMD is cleaved by caspase (classic and noncanonical pyroptosis pathway) and subsequently integrated into the cell membrane (72). Caspases also activate inflammasomes that cleave inflammatory cytokine precursors to release IL-1β, IL-18, and high mobility group box 1 (HMGB1) (72). Wang et al. discovered that Mg^2+^ can shield against LPS-induced sepsis by impeding GSDMD N-terminal-induced pyroptosis (73). HMGB1 triggers further pyroptosis by re-transporting LPS into the cell via the receptor of advanced glycation end-products, thereby creating an inflammatory loop (49). Researchers have been focusing on the inhibition of canonical pyroptosis in neutrophils and monocytes/macrophages by NOD-, LRR-, and pyrin domain containing 3 (NLRP3) inflammasome, which could rescue lethal sepsis in different septic animal models (74). Nowadays researchers have also focused on lymphocytes and found that inhibition of T cell pyroptosis could increase survival after lethal CLP-induced sepsis (75). Over pyroptosis has been demonstrated to promote the aggravation of sepsis. Therefore, effective and specific blockade of cellular pyroptosis may be a valuable therapeutic intervention to rescue sepsis.

### 3.3 Autophagy

Autophagy is a self-defense mechanism for cell survivorship, forming double-membrane autophagosomes and degrading substrates such as compromised organelles and pathogens (76). In the hyperinflammatory phase of sepsis, autophagy exerts a protective function by eliminating pathogens, neutralizing toxins, preserving mitochondrial integrity, and regulating cytokine secretion (76). However, autophagy efficiency reduction or procedure disturbances may cause damage. For example, the blockade of CD4^+^ T cell autophagy is associated with increased IL-10 production. Additionally, autophagy-related gene 4B- deficient mice exhibit high mortality and severe ALI in response to LPS stimulation (76). Impaired autophagic flux accumulates high levels of autophagosomes and can't recycle damaged mitochondria (77). Furthermore, the overproduction of outer membrane vesicles causes excessive activation of the inflammasome, resulting in the destruction of autophagosome-lysosome fusion (78). Autophagy in non-immune cells has also been associated with sepsis. The preservation of endothelial cell permeability depends on the stabilization of adherent junctions and endothelial barriers through complete autophagy, thus reducing sepsis-related endothelial damage (79). Therefore, curative activation of complete autophagic flux may be an effective target for sepsis (76).

### 3.4 Ferroptosis

Ferroptosis is the iron-dependent lipid peroxidation of cell death. Ferroptosis is involved in LPS-injected ALI (80). Ferroptosis is initiated in immune cells due to an imbalance in the antioxidant system of glutathione peroxidase 4 (GPX4), leading to an increase in lipid peroxide levels (81). Thus, the ferroptosis program not only provides nutrients to bacteria but undoubtedly reduces the immune function of hosts. Regulation of antioxidant molecules (e.g., GPX4, glutathione) and targeting the regulatory pathways of ferroptosis could be a new approach for the treatment of sepsis (81).

### 3.5 Crosstalks between cell death modes

The effects of the various forms of cell death caused by sepsis are not independent. Autophagy deficiency could lead to immune cell dysfunction and accelerated apoptosis, resulting in reduced immunity and increased mortality in septic mice (77). Mitochondrial autophagy inhibits pyroptosis by reducing the production of reactive oxygen species (ROS), also known as NLRP3 activators (74). The induction of ferroptosis in pulmonary epithelial cells by NETs has been shown to exacerbate CLP-induced ALI in mice, mechanically through the implementation of methyltransferase-like 3-induced m6A modification of GPX4 (82). Both pyroptosis and ferroptosis participate in CLP-induced ALI in mice, which is induced by ROS (83). Apoptotic caspase-7 cleaved GSDMB (a potential proinflammatory mediator in sepsis) to block non-canonical pyroptosis to avoid over-inflammation in sepsis (84). PANoptosis encompasses the overlapping and interdependent mechanisms of pyroptosis, apoptosis, and necroptosis (85). PANoptosome serves as a death complex, a multi-protein molecular framework that recruits downstream inflammatory cell death components such as NLRP3 inflammasome, the RIPK1/RIPK3 complex, and more (86). Moderate PANoptosis plays a crucial role in efficiently scavenging infectious agents and activating immune cells. However, excessive PANoptosis may result in tissue damage. Therefore, achieving a balanced regulation of this process represents a critical direction for future research into PANoptosis (85). The interplay and equilibrium among several cell death patterns remain a challenge for future research.

## 4 Organs in sepsis

Recently, researchers utilized spatial transcriptomics to measure gene expression in 9 organs following sepsis, creating a dynamic, organism-wide map. Interestingly, they verified pairwise cytokines' effects on nearly 200 cells and found that non-lymphoid tissues recover to transcriptional homeostasis earlier than lymphoid tissues. This may help explain why post-septic patients are in a state of prolonged immunosuppression (87). We reviewed several important organs and their role in sepsis.

### 4.1 Gut

Gut is the main source of infection, leading to secondary strikes on sepsis survivors, hindering the immune system, and elevating readmission rates and mortality (88). Metabolites with anti-inflammatory effects such as short-chain fatty acids and Granisetron decrease, and Granisetron reduces CLP-induced-septic damage of the gut-liver axis (89, 90). Colonization of pathogenic and opportunistic pathogens in the gut takes a dominant position but the growth of commensal organisms was depressed, resulting in the disruption of the immune barrier and the reduction of beneficial metabolites (91). Regeneration of intestinal barrier cells is inhibited and apoptotic cells increase during sepsis, with a reduction in luminal coverage, thickness, and adhesion. Inflammatory cytokines upregulate claudin 2 and junctional adhesion molecules but downregulate claudin 5 and zonula occludens-1, rendering the intestinal hyperpermeability (92). In conclusion, the mechanical, immune, and biological barriers of the septic gut are disturbed after sepsis. The massive proliferation of pathogens and endotoxins could be transferred to the systemic circulation via the mesenteric lymphatics or portal vein (93). Our viewpoint is focusing on the importance of gut among multiple organs, because many other organ axes are associated with the gut. The gut may predispose to more severe secondary infections in septic survivors.

### 4.2 Liver

Both biliary and portal circulations continuously defend against invading bacteria. Pathogens can impair liver function, primarily by reducing bacterial clearance, disrupting detoxification, and interfering with the release of inflammatory cytokines during sepsis (94). When the intestinal barrier is compromised, pathogen-associated molecular patterns and damage-associated molecular pattern molecules intrude via two circulations into the liver, where they recognize pattern recognition receptors on Kupffer cells (KCs) or stellate cells, resulting in inappropriate immunogenicity initiation or overwhelming inflammation response (95). One of the features of the immunogenic response is an increase in acute phase proteins (APPs) and the induction of immunosuppression and ET associated with MDSCs by APPs (95). Sepsis modulates the polarization of KCs towards the M1 phenotype, which contributes to the immunoinflammatory response and acute liver injury (96). Peng et al. found that pharmacological inhibition of M1 polarization could alleviate LPS/D-galactosamine-induced liver injury in mice (97). Sepsis-induced cholestasis commonly results in impaired hepatic glycolipid metabolism and apoptotic necrosis (96). Decreased bile acids in the enterohepatic circulation also facilitate intestinal bacterial translocation that increases mortality (98).

### 4.3 Lung

The lung is particularly susceptible to damage from sepsis, with approximately half of septic patients developing acute respiratory distress syndrome (ARDS) in the later stage, leading to a further increase in patient mortality (94). Notably, the presence of gut-associated microbes has been demonstrated in the lung of septic patients (99). Carbapenemase-producing Enterobacteriaceae intestinal colonized mice have a notable decrease in pulmonary alveolar macrophages and DCs, and the outcome after secondary Pseudomonas aeruginosa lung strikes is worsened (100). Dickson et al. showed that enterobacteria can metastasize across the intestinal mucosa and even into the lungs in septic-ARDS patients, as verified by analysis of the ecology of lung-associated bacteria in both LPS and CLP models of sepsis (99). The "mesenteric lymphatic hypothesis" has also been supported by multiple lines of evidence. Transfer of gut lymph from critically ill mice to healthy mice causes lung injury (101). Consequently, the attenuation of injury associated with the gut-lung axis in ARDS merits further investigation.

### 4.4 Bone marrow

BM serves as the primary lymphoid organ and provides a microenvironment for hematopoietic stem and progenitor cells (HSPCs). A humanized mice model stimulated with CLP/endotoxemia induces HSPCs expansion with a concomitant shrinkage of the downstream progenitor cells in the BM (27). Wang et al. found that the frequency of HSPCs in the peripheral blood of septic patients on day 4 after sepsis is significantly higher than healthy controls (*P* < 0.05) (102). The expansion of HSPC at early time points in septic patients had a negative correlation with immune cell counts (*P* < 0.05) (102). Through subpopulation analysis, the most pronounced change in HSPCs is the common myeloid progenitors (CMPs) (102). Deviation of CMPs from normal differentiation to pathological activation may increase the population of peripheral blood CD34^+^ CD38^+^ HSPCs. Sepsis-induced BM niche with ablation of osteoblasts associated with reduction of common lymphoid progenitor cells and dysfunction of hematopoietic stem cells (64). Lymphopenia may also be partly caused by the decline in common lymphoid progenitor cells. Macrophage function is impaired partly due to the epigenetic modification of BM progenitor cells with lower expression of mixed lineage leukemia protein-1 (103). Disruption of the homeostatic balance of self-renewal and differentiation of HSPCs ultimately leads to depletion of the progenitor compartment. The infusion of donor HSPCs to a group A Streptococcus-induced sepsis mouse model has increased overall survival (104).

### 4.5 Spleen

As the largest peripheral immune organ, T-cell exhaustion and suppressor cell expansion in the spleen may present important immunosuppressive mechanisms in sepsis (105). Splenic macrophages in red marrow and marginal zone express CD169 and the receptor SIGN-related 1. The CD169 macrophages disrupt neutrophil antimicrobial function, deplete mature neutrophils and promote T cell apoptosis, ultimately leading to a reduction in both innate and adaptive immunity in systemic candidiasis (106). Spleen enriches the CD40L ligand platelet population during cecal slurry injection-induced sepsis in mice, potentially facilitating improved bacterial clearance (possibly via increased NETs), reducing organ injury, and improving the survival of mice (107). Various cell subpopulations of the spleen exert different influences in sepsis. Thus, the hematopoietic and immune functions of the spleen in sepsis merit further exploration.

### 4.6 Central nervous system

The central nervous system (CNS) modulates the immune response and is responsible for the collapse of the peripheral immune system. Neuroimmune reflexes such as the vagus nerve pathway, hypothalamic-pituitary-adrenal (HPA) axis, and central circadian system are disturbed by sepsis, resulting in extensive loss of neurons and cognitive disorders. The formed vicious cycle between sepsis-associated encephalopathy and turbulent immunity leads to poor outcomes in septic patients (108). Constitutive activation of the vagus nerve after CLP in mice blocked the release of TNF-α and was incompetent to the second LPS challenge, and the α7 nicotinic acetylcholine receptor plays an important role in sepsis neural regulation (109). The anti-inflammatory HPA axis inhibits the production of proinflammatory cytokines and reaches ephemeral endogenous glucocorticoids peak in sepsis (108). However, long-stay ICU patients with suppressed HPA axis suffer central (secondary) adrenal insufficiency (110). The target tissues of many septic patients show resistance to glucocorticoids and fail to maintain immune tolerance (111). The suprachiasmatic nucleus modulates the cyclic expression of circadian clock gene programs. It was demonstrated that the core biological clock gene Bmal1 trans-regulates immune checkpoint PD-L1 on macrophages and T-cell apoptosis in CLP-induced sepsis (112). Similarly, mice deficient in Per2 are more resistant to LPS-induced septic shock. The CNS is a major predecessor in the regulation of the release of the inflammatory cytokines. Dysregulated hormonal secretion rhythms and immune system disorders occur in the early stages of sepsis. Most of the immune cells may express catecholamine receptors and rhythmic genes, so maintenance of CNS is necessary to control the immune imbalance.

## 5 Epigenetic and metabolic reprogramming in sepsis

Epigenetic modifications activate or deactivate transcriptional regulation without altering the DNA sequence, and have been proven to regulate sepsis ER (8). DNA methylation, non-coding RNA, and histone modifications within immune cells contribute to the pathophysiological mechanism of sepsis. Histone H3 lysine 9 dimethylation in late sepsis inactivates the promoter of TNF and IL-1β transcription, downregulating immune sensitivity to endotoxin stimulation (111). The high dynamics of histone deacetylases (HDACs) overwhelm the acetylation of histone acetyltransferases (HATs) to progress immunosuppression and HDAC inhibitors have been shown to improve sepsis progression. Panobinostat as a pan-HDAC inhibitor could inhibit IL-10 production in LPS-stimulated macrophages (113). Although HDAC inhibitors have been clinically approved, it remains to be elusive to locate the precise timing to intervene in gene silencing during immunoparalysis. DNA methylation has a more precise and stable function than histone modifications, connected with the stabilization of the transcriptional state. Binnie et al. aimed to identify significant DNA methylome change in tolerized monocytes in septic patients and highlighted that DNA methylation is determined by the altered levels of inflammatory cytokines (114). Other researches also demonstrate that DNA methylation is involved in monocyte dysregulation during sepsis. The absence of RNA m6A demethylase alkB homolog 5 in neutrophils inhibits mRNA expression of chemokine receptor CXCR2, thus injuring the neutrophil migration, as observed in CLP-induced mouse model of sepsis (115). MiR-221, miR-579, and miR125b tend to directly prevent TNF translation for the existence of binding sites for the 3’ untranslated region of TNF, while miR146a acts indirectly (116). The increased expression of specific microRNAs (e.g., miR-220, miR-221, miR-222) in macrophages correlates with immunoparalysis in septic patients and serves as biomarkers of immunosuppression (117). MiR-210 coordinates M1 macrophages and increases glycolysis in response to LPS stimulation at the expense of oxidative phosphorylation (OXPHOS) (117). Epigenetic modification agents show promise as therapeutic tools in animal models of sepsis (118). With the numerous mechanisms already discussed, epigenetic regulation occupies the center of the cellular immune metabolism and immunologic responses of sepsis.

The altered metabolic pathways of immune cells and the abnormal accumulation of the metabolites in sepsis both play vital regulation roles. Immune cells convert from OXPHOS to glycolysis as the main metabolic source to rapidly produce high quantities of ATP and activate the immunological effect during sepsis (119). However, patients in the late stage presented mitochondrial dysfunction and oxidative imbalance. This has been demonstrated by a reduction in cytokine production and impaired phagocytosis in PBMCs with immune tolerance (119, 120). Leukocytes in the immunosuppression phase of sepsis present extensive immunometabolic defects, with impaired glycolysis and oxidative metabolism (120). It is promising that the immunostimulatory therapy, recombinant IFN-γ, has been demonstrated to partially reverse metabolic paralysis in septic patients, and it can promote polarization of macrophages towards the M1 phenotype (120). Intact mitochondrial complexes are integral to support the long-term survival and functions of macrophages (121). A recent study has indicated that mitochondrial STAT3 drives FAO to worsen sepsis in LPS-injected mice (122). Mitochondrial dysfunction in sepsis is unable to provide adequate biological energy and associated with unfavorable clinical endpoints (121).

LPS stimulation compromises macrophage activity and results in high glycolysis and abnormal accumulation of tricarboxylic acid (TCA) cycle intermediates (120). Pyruvate kinase M2 (PKM2) is upregulated in activating macrophages and the tetrameric form of PKM2 resists tyrosine phosphorylation, which attenuates glycolytic biosynthetic intermediates accumulation. The tetramer form prevents nuclear translocation-induced transcription of hypoxia-inducible factor-1α (HIF-1α) and IL-1β, but promotes M2 phenotype and ET (123). As a glycolytic key metabolite, PKM2 not only promotes the release of HMGB1 in sepsis, and its inhibition also decreases the production of another vital metabolite, lactate.

Lactate has been considered as a metabolic waste, but in recent years lactate has been discovered as signaling molecule, biomarker, and modifier of lactation in sepsis. High lactate induces the release of HMGB1 into circulation by lactylation/acetylation via exosome to disrupt endothelium integrity and vascular permeability in CLP-induced septic mice (124). Lactate overproduction is correlated with the high morbidity and poor prognosis of septic patients with acute kidney injury (AKI). The results have shown that lactylation at lysine 20 of Fis1 may be the mechanism of lactate to cause CLP-induced AKI (123). High circulation lactate levels promote the inactivation of immune cells and the production of immunosuppressive cells. Hyper-lactate also increases the expression of anti-inflammatory genes TGF-β and IL-10, promoting M2 macrophage generation and inhibiting DC differentiation (121).

Itaconate is a macrophage immunometabolite with anti-inflammatory and immunomodulatory properties after sepsis (125). The main effects of itaconate are the anti-oxidative stress pathway of nuclear factor E2-related factor 2 (Nrf2) as well as the influence on succinate dehydrogenase (SDH) and NLRP3 inflammasomes (125, 126). Dimethyl itaconate (itaconate derivative) ameliorates survival within 24h and reduces ALI in septic mice (127). And 4-OI reduces inflammatory cytokine and lactate production in mice exposed to lethal endotoxemia to prolong survival (128). Nrf2 is generally known to control oxidative stress by mediating glutathione synthase. In CLP-induced sepsis in mice, Nrf2 may also function as a transcriptional suppressor to inhibit PD-L1 expression (129). Researchers have discovered an additional facet of itaconate's function. Wu et al. found that the CDK2-aconitate decarboxylase 1 axis triggers the inflammatory cytokines storm, with its expression levels correlating with sepsis severity in patients (130). Similarly, Chen et al. found that MYD88-STING1-mediated itaconate production exacerbates CLP- and LPS-triggered sepsis (131). Interestingly, β-glucan bypasses the inhibition of SDH by itaconate and reconstitutes macrophage metabolism and immunity (132). Therefore, we hypothesize that the reductions in itaconate production could restore TCA circulation activity, thereby improving immune tolerance. Currently, we are uncertain whether itaconate is favorable for the restoration of metabolic paralysis in sepsis and it deserves further investigation (132).

## 6 Artificial intelligence and biomarkers in sepsis

No universally accepted gold standard was conceded for the treatment of sepsis during the past years. With the development of bioinformatics, artificial intelligence is combined with disease diagnosis and prediction. Machine learning (MI) is trained to build more accurate models on medical data and assist clinicians in the early classification and diagnosis of sepsis (133). In contrast to the algorithms built on structured data, the SERA algorithm has extracted and synthesized unstructured data from electronic medical records, and the algorithm predicted sepsis 12 hours in advance with high sensitivity and specificity (AUC = 0.94) (134). MI is not just to accurately predict and diagnose sepsis. MI is also used to make antibiotic recommendations for sepsis. After training and validation with two intensive care testing sets ( the MIMIC-III and AmsterdamUMCdb), the antibiotic treatment strategy recommended by the T4 model is proven to be effective in decreasing the mortality rate (135). The COMPOSER model was trained for the early identification of patients with a high risk of sepsis and abandoned the prediction of false predictions to improve accuracy (AUC = 0.925–0.953) (136). Recently this model was identified to be associated with an increase in the bundle compliance of sepsis (137).

Hyperinflammation and immunosuppression fail to precisely characterize the mechanisms for disease progress in septic patients. MI with multi-omics databases would classify patients into subgroups within similar pathological dynamics (133). Group-based trajectory modeling (GBTM) was utilized to model the evolution of physiology repeated-measured temperature trajectory with septic patients to classify sub-phenotypes. Four temperature trajectory groups were identified. The investigators speculated on the association of immunological phenotypes with temperature subtypes, with hypothermic subjects being possibly more relevant to immunosuppression (138). MI quantified similarities between septic individuals and differentiated patients into phenotypes basing not only on temperature or organ dysfunction, but also fluid responsiveness (139). However, the most prevalent technique is to deeply mine whole blood immune cell transcriptomics with MI (**Table 1**) (133). How to downsize multiple dimensions and integrate sepsis heterogeneity are future challenges, the key biomarkers might fill the vacuum of the clinical and transcriptomic data (144). The ideal biomarker should meet the following criteria: accurate identification, prognosis prediction, and easy detection. However, individual biomarkers may not be fully satisfied. The combined application of biomarkers may compensate for the limitations of diagnostic specificity or sensitivity and potentially yield higher diagnostic value. Most high inflammatory biomarkers are used to diagnose early sepsis, while immunosuppressive biomarkers can predict early or late prognosis in septic patients. The challenge lies in correctly combining the appropriate biomarkers to achieve optimal diagnostic results. **Tables 2**, **3**

**Table 1:** Machine learning models for sepsis phenotypes.

Machine learning models	Classifier (Algorithms)	Data source	Stratifications	Main clinical observations (Efficiency)	References
**LPA**	Clinical variables, Platelet count, creatinine, mean blood pressure, arterial partial oxygen pressure, etc. (LPA)	Data about 14,993 patients from MIMIC- III database	Profile 1, low mortality outcome.		(140)
			Profile 2, respiratory dysfunction.	Associated with increased risk of 90-day mortality (HR = 1.15)	
			Profile 3, multiple organ dysfunction involving kidney, liver, coagulation, and circulatory failure.	Associated with increased risk of 90-day mortality (HR = 1.79) and increased risk of hospital morality (OR = 2.16)	
			Profile 4, neurological dysfunction.		
**Temperature trajectories model**	Temperature trajectory measurements within the first 72 hours (GBTM)	The temperature trajectories of 12413 patients who received antibiotics within 24 hours of presentation	Hyperthermic, slow resolvers, including the youngest patients and had the fewest comorbidities, the highest levels of inflammatory markers	Highest in-hospital mortality rate of 10.2% (*P* < 0.001)	(139)
			Hyperthermic, fast resolvers	The lowest mortality risk (OR = 0.55, *P* < 0.001)	
			Normothermic		
			Hypothermic, including the oldest patients and had the most comorbidities, the lowest levels of inflammatory markers.	Highest in-hospital mortality rate of (*P* < 0.001)	
**Clinical phenotypes**	29 candidate variables, demographic variables, vital signs, systolic blood pressure, temperature, and oxygen saturation), hemoglobin, chloride, bicarbonate, lactate, and albumin, etc.	Data about 20189 septic patients at 12 Pennsylvania hospitals from 2010 to 2012	α, patients with the lowest administration of a vasopressor.		(141)
			β, patients are older and had more chronic illness and renal dysfunction.		
			γ, patients had more inflammation and pulmonary dysfunction.	Highest endothelial dysfunction markers (*P* < 0.01)	
			δ, patients had more liver dysfunction and septic shock.	Highest 28-day and 365-day mortality (*P* < 0.001).	
**MARS**	A 140-gene expression classifier from whole-blood RNA expression profiles	306 septic patients from two ICUs in the Netherlands between 2011 and 2012	Mars1, BPGM and TAP gene expression.	Highest 28-day mortality of 39% (HR = 1.86, *P* < 0.0045)	(142)
			Mars2, GADD45A and PCGF5 gene expression.		
			Mars3, AHNAK and PDCD10 gene expression.		
			Mars4, IFIT5 and GLTSCR2 gene expression.		
**SRS**	The peripheral blood leucocyte global gene expression	265 septic patients admitted to ICU	SRS1, immunosuppression with endotoxin tolerance, T-cell exhaustion, and downregulation of human leucocyte antigen class II.	Higher 14-day, 28-day and 6-month mortality (HR = 2.4, *P* < 0.005)	(142)
			SRS2, relatively immunocompetent.		
**Deep learning-based clustering**	5-gene class model, C14orf159、AKNA、PILRA、STOM and USP4 (genetic algorithms)	The whole blood gene expression profiling in adult sepsis patients from GEO and ArrayExpress databases	Class 1, immunosuppression	Higher mortality of 21.8% (*P* < 0.01)	(143)
			Class 2, relatively immunocompetent.		
**Organ dysfunction trajectory model**	72-h SOFA score trajectories (DTW and HAC)	4678 septic patients from MIMIC-III database to develop model	Rapidly Worsening, continuously increased SOFA scores from a mean of 4.5 at admission to more than 7 at 72h.	Highest in-hospital mortality of 28.3% (*P* < 0.001)	(143)
			Delayed Worsening, decreased SOFA scores within the first 48h from a mean of 5.2 at baseline to 3.7, followed by an increase over the last 24h.		
			Rapidly Improving, a consistent continuous improvement in SOFA scores from a mean of 5.54 at baseline to less than 3.	Lowest rate of mortality of 5.5% (*P* < 0.001)	
			Improving, an increase and then a gradual decrease in SOFA score over 72h.		

LPA, latent profile analysis; HR, hazard ratio; OR, odd ratio; GBTM, group-based trajectory modeling; MARS, molecular diagnosis and risk stratification of sepsis; ICU, intensive care unit; BPGM, bisphosphoglycerate mutase; TAP2, transporter 2, ATP binding cassette subfamily B member; GADD45A, growth arrest and DNA damage inducible alpha; PCGF5, polycomb group ring finger 5; PDCD10, programmed cell death; IFIT5, interferon induced protein with tetratricopeptide repeats 5; GLTSCR2, glioma tumour suppressor candidate region gene 2; SRS, sepsis response signature; GEO, gene expression omnibus; DTW, dynamic time warping; HAC, hierarchical agglomerative clustering; MIMIC-III, Medical Information Mart for Intensive Care III database.

**Table 2:** Biomarkers for immunosuppression in sepsis.

Biomarkers	Main pathophysiologic role	Cut-off value	Observation endpoints (Efficiency)	References
**Persistent** **lymphopenia**	An independent predictor of adverse clinical outcomes in sepsis	<1.0 × 10^9^/L (on day 4)	Predict 28-day mortality (AUC = 0.68, sensitivity 76% and specificity 56%)	(44)
		≤0.6 × 10^9^/L (on and after day 4)	Increased development of secondary infections (*P* = 0.04)	
**NLR**	A commonly detected indicator that often rises during sepsis	> 4.18–31	Poorer prognosis for adult (HR = 1.6884)	(145)
			Predict early mortality (AUC = 0.8, sensitivity 64% and specificity 79%)	
**M-MDSCs**	An immature cell population with immunosuppressive activity	> 9.1% among total monocyte population at day 6–8	Predict 28-day mortality (OR = 4.4, *P* = 0.001)	(63)
			Associated with high hospital-acquired infection (OR = 2.4, *P* = 0.013)	
**Th2/Th1**	Th1 cells release proinflammatory cytokine Th2 cells secret anti-inflammatory cytokine	> 2.74 on day 7	Predict 28-day mortality (AUC = 0.869, sensitivity 66.1% and specificity 92.5%)	(47)
		> 2.95 on day 3	Predict 28-day mortality (AUC = 0.831, sensitivity 75% and specificity 95.1%)	
		Non-recovery ratio of Th2/Th1	Highest mortality (47.1%) and higher incidence of ICU-acquired infections (64.7%, *P* = 0.012)	
**mHLA-DR**	A marker of monocyte anergy and deactivation	Mean Fluorescent Intensity ≤ 54 on day 3–4	Independently associated with nosocomial infections after sepsis (AUC = 0.65, sensitivity 68% and specificity 62%) (AHR = 2.52, *P* = 0.02)	(28)
		ΔmHLA-DR_7_ ≤ 9%	Predict 28-day mortality (AUC = 0.938, sensitivity 85.7% and specificity 90%)	(146)
**sPD-1/sPD-L1**	Immune checkpoint	sPD-1 ≥ 17.7 pg/mL	Diagnose sepsis (AUC = 0.74)	(147)
		sPD-L1 ≥ 29.9 pg/mL	Diagnose sepsis (AUC = 0.77)	
**IL-10**	A pleomorphic cytokine	> 3.05 pg/mL	Independent predict mortality (AUC = 0.942, *P* = 0.0001, sensitivity 91.67% and specificity 86.96%)	(29)
			Early diagnosis of bacterial SlRS (AUC = 0.767, *P* = 0.0021, sensitivity 78.26% and specificity 80%)	

AUC, area under curve; NLR, neutrophil-to-lymphocyte ratio; HR, hazard ratio; M-MDSCs, monocytic myeloid-derived suppressor cells; OR, odd ratio; Th1, T helper type 1; Th2, T helper type 2; ICU, intensive care unit; mHLA-DR, monocyte human leukocyte antigen-DR; AHR, adjusted hazards ratio; sPD-1, soluble programmed death-1; sPD-L1, soluble programmed cell death ligand 1; IL-10, interleukin 10; SlRS, systemic inflammatory response syndrome.

**Table 3:** Biomarkers for pro-inflammation in sepsis.

Biomarkers	Main pathophysiologic role	Cut-off value	Observation endpoints (Efficiency)	References
**CRP**	A pentameric protein induced by inflammatory cytokines and produced by the liver	> 12 to 90 mg/L	Diagnosis sepsis (AUC = 0.73, sensitivity 80% and specificity 61%)	(148)
**PCT**	A precursor of calcitonin produced by C-cells of the thyroid gland	> 0.28 to 4.5 ng/mL	Diagnosis sepsis (AUC = 0.84 sensitivity 80% and specificity 75%)	(149)
		< 0.5 ng/mL to stop antibiotic therapy	Associated with lower 28-day mortality (OR = 0.84, *P* = 0.008)	(149)
			Shorter antibiotic therapy (MD = 1.79, *P* < 0.001)	
**IL-6**	A universally recognized biomarker for early sepsis detection and early phase pro-inflammatory cytokine	> 35 to 620 pg/mL	Diagnosis sepsis (AUC = 0.81, sensitivity 73% and specificity 76%)	(150)
**CD64**	An Fcγ receptor elevating rapidly after infection on monocytes	Different measurement methods	Diagnosis sepsis (AUC = 0.94, sensitivity 87% and specificity 89%)	(151)
**Presepsin**	A soluble subtype of CD14	> 101.6 to 1000 pg/mL	Diagnosis sepsis (AUC = 0.87, sensitivity 84% and specificity 73%)	(148)
**Calprotectin**	A calcium-binding protein that originally found in neutrophils	> 1300 to 38300 ng/mL	Diagnosis sepsis (AUC = 0.88, sensitivity 77% and specificity 85%)	(152)
**sTREM-1**	A member of immunoglobulin family predominantly expressed on the neutrophils and monocytes and upregulated in infection	> 30 to 60 ng/mL	Diagnosis sepsis (AUC = 0.88, sensitivity 82% and specificity 81%)	(153)

CRP, c-reactive protein; AUC, area under curve; PCT, procalcitonin; OR, odd ratio; MD, mean difference; IL-6, interleukin 6; sTREM-1, soluble triggering receptor expressed on myeloid cells-1.

## 7 Conclusions and future perspectives

We have profiled the mechanism of sepsis from multiple aspects. Hyperinflammation in sepsis is accompanied by prolonged immunosuppression. Excessive immunosuppression is the foundation of PICS, high readmission rates, and increased secondary mortality. Owing to the heterogeneity of sepsis, clinicians can’t treat patients with “one-size-fits-all” therapy. The establishment of a therapeutic regime for sepsis would require an investigation into the mechanisms of the host immune disorder, in particular the intricate network among epigenetic alterations and metabolic reprogramming, and other mechanisms. Primarily, researchers require the identification of a uniformly approved set of biomarkers, which is accessible and easily testable. Secondly, the standardized sepsis subgroups and endotypes could help clinicians identify patients who develop immunosuppression. Finally, personalized precision therapy potentially could be based on artificial algorithms which in anticipation would dynamically mimic the course of septic patients. Overall, continued research in sepsis has the potential to greatly improve our understanding and treatment of this devastating condition.

## Author contributions

XZ: Writing – original draft. YZ: Writing – original draft. SYY: Conceptualization, Funding acquisition, Writing – review & editing. JZ: Conceptualization, Funding acquisition, Writing – review & editing.
